# Absolute Quantification and Spatial Mapping of Hyaluronic Acid in Histological Tissue Sections

**DOI:** 10.1021/acsmeasuresciau.5c00138

**Published:** 2025-11-11

**Authors:** Cristina Quílez, Jorge González-Rico, María Luisa López-Donaire, Nuria Gago-López, Arrate Muñoz-Barrutia, Diego Velasco

**Affiliations:** † Bioengineering Department, Universidad Carlos III de Madrid, 28911 Leganés, Spain; ‡ 16726 Fundación Instituto de Investigación Sanitaria de la Fundación Jiménez Díaz, 28040 Madrid, Spain; § Continuum Mechanics and Structural Analysis Department, Universidad Carlos III de Madrid, 28911 Leganés, Spain; ∥ Melanoma group, Molecular Oncology Program, Spanish National Cancer Research Center (CNIO), 28029 Madrid, Spain; ⊥ Neuroscience and Biomedical Sciences Department, Universidad Carlos III de Madrid, 28903 Getafe, Spain; ∇ Instituto de Investigación Sanitaria Gregorio Marañón, 28009 Madrid, Spain

**Keywords:** Hyaluronic acid, quantification, fluorescence, paraffin, tissue sections

## Abstract

We present an accessible methodology for the absolute quantification and spatial mapping of hyaluronic acid (HA) in paraffin-embedded tissues. Although HA plays critical roles in tissue hydration, organization, and disease progression, its local concentration and spatial distribution remain poorly defined due to the lack of quantitative measurement tools. By integrating immunofluorescence, ELISA, and image analysis our approach enables the generation of pixel-level HA concentration maps. This strategy bridges the gap between qualitative imaging modalities and bulk biochemical assays, offering both spatial resolution and quantitative data. The method is validated across multiple tissues and is robust, scalable, and readily applicable to other extracellular matrix components, offering a practical tool for studying tissue microenvironments in health, disease, and biomaterial design.

## Graphical abstract

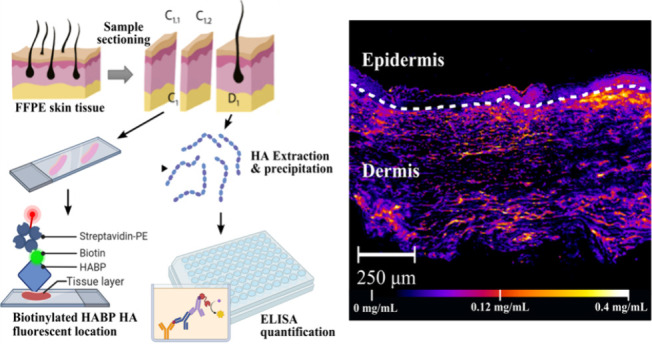

The extracellular matrix (ECM) provides structural and biochemical signals that guide tissue-specific cell behaviors.^1^ Its composition—comprising collagens, proteoglycans, laminins, and fibronectin—is uniquely structurally organized in each tissue and dynamically regulated during development, homeostasis, and disease.^2^ A key ECM component is hyaluronic acid (HA), a nonsulfated glycosaminoglycan (GAG) abundant in soft connective tissues.^3^ Hyaluronic acid (HA) plays key roles in hydration, tissue organization, angiogenesis, inflammation, and cell migration,^4^ all of which are essential for both normal physiological processes and disease progression.^5,6^ The biological activity of HA is strongly influenced by its molecular weight and concentration,^7^ which have been associated with cancer type and prognosis^8^ as well as neurodevelopmental processes^9^ and synaptic function.^10^ Due to its multifunctional nature and ubiquitous presence, HA has also emerged as a promising biomaterial in tissue engineering, particularly for creating three-dimensional cell culture scaffolds that closely mimic the native ECM.^11^ However, establishing HA as a complementary diagnostic marker^12^ or effectively replicating its functions in engineered tissues requires a more comprehensive understanding of its in vivo concentration and spatial distribution—parameters that remain poorly characterized. Addressing these knowledge gaps may not only clarify the role of ECM-associated HA but also facilitate its broader application as a scaffolding molecule and disease biomarker. Several approaches have previously been used to visualize or estimate HA distribution in tissues, including histochemical staining, HA-binding protein-based fluorescence, and biochemical extraction coupled with imaging readouts.^13–17^ More recently, mass spectrometry (MS)-based methods such as Matrix-Assisted Laser Desorption/Ionization (MALDI) imaging have also been applied.^18^ Although innovative, this method is complex and limited in spatial resolution. Furthermore, the scope of the study lies uniquely on one set of *ex vivo*-obtained skin samples. This is due to its high complexity and specificity. While these strategies have provided valuable insights, they remain largely semiquantitative and often lack reproducibility or spatial resolution at the pixel level. In contrast, the methodology presented in this work establishes a calibration pipeline that combines HA-based fluorescence imaging with ELISA measurements from paired sections, enabling absolute quantification of HA concentration maps in paraffin-embedded tissues. This integration represents a practical advance beyond existing semiquantitative approaches, allowing reproducible, pixel-level evaluation of HA distribution across diverse tissue types. Furthermore, the method proposed in this work can be generalized to multiple sample types, taking advantage of the ubiquitous presence of HA across different tissues and species.

For this study, human, rat and mouse skin, brain, and liver biopsies of 2–3 cm^2^ were fixed using formalin-free tissue fixative (A5472, Sigma-Aldrich, USA) to prevent HA loss.^19^ Then, tissues were embedded in paraffin (see the Supporting Information (SI)) and sectioned in a sequence of two 5 μm thickness samples (*C*_*n*__.*m*_) used for fluorescence staining followed by a 400 μm thickness sample (*D*_*n*_) used for tissue digestion (Figure 1A). These tissues were chosen to account for a broad spectrum of HA concentration as reported in the literature.^13^ The fluorescence staining of HA within tissue sections started with the deparaffinization of the *C*_*n*__.*m*_ samples (Figure 1B, SI). Next the unspecific binding sites of the tissues were blocked using 3% Phosphate-Buffered Saline (PBS) - Bovine Serum Albumin (BSA). For HA fluorescence staining, the samples were incubated first with Biotinylated Hyaluronic Acid Binding Protein (HABP-b) (AMS.HKD-BC40, AmsBio, USA), followed by secondary probe staining, with Alexa Fluor 488-Streptavidin conjugate (S11223, ThermoFisher, USA) (SI).

**Figure 1:**
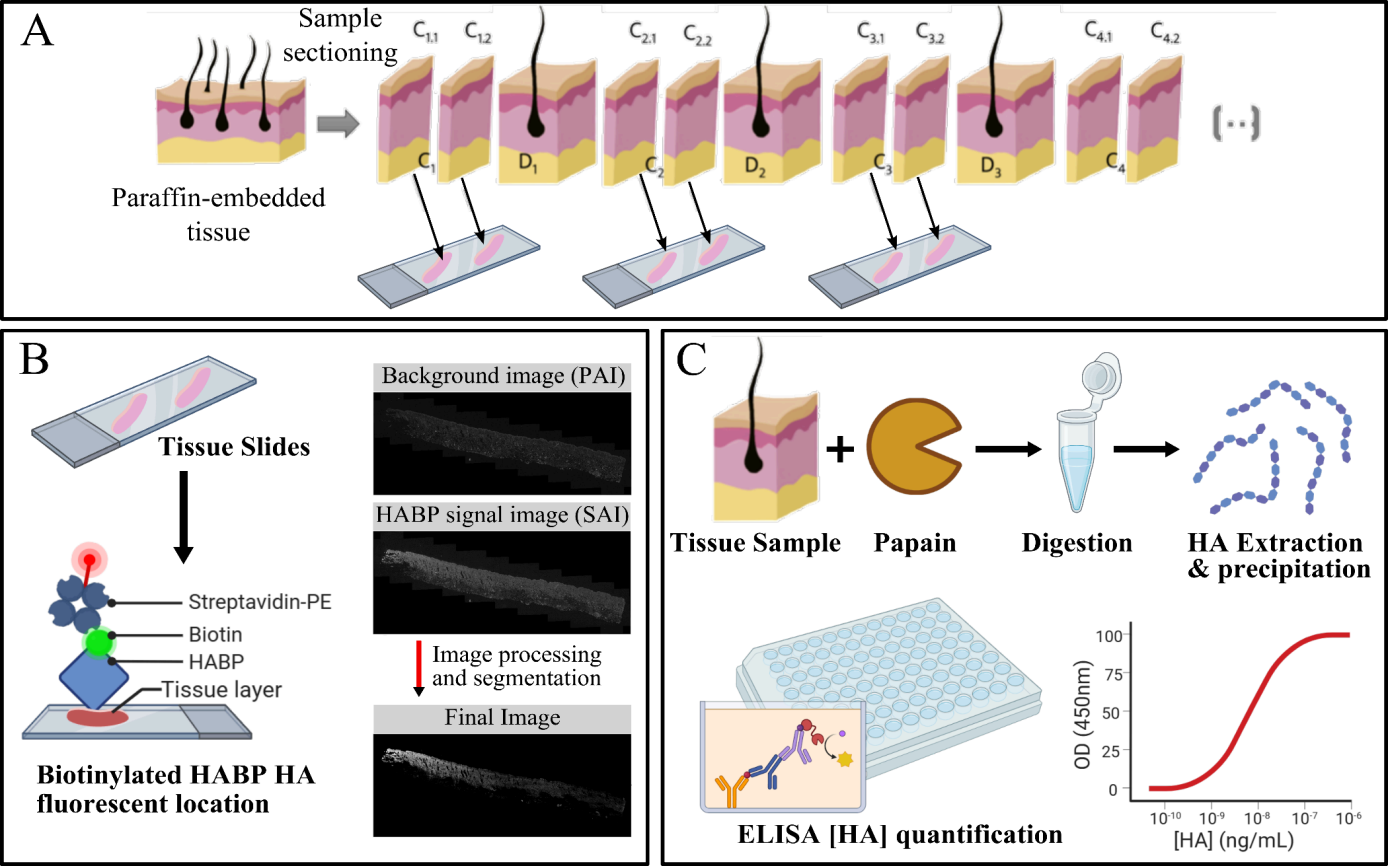
Sample processing and analysis to obtain a correlation of the FI signal and HA concentration for the generation of quantitative HA distribution. (A) Schematics showing the methodology for the sectioning procedure for paraffin-embedded samples. (B) The 5 μm cuts (*C*_*n*.1_, *C*_*n*__.2_, ...) were placed on top of Polysine slides to be stained with Hyaluronic Acid Binding Protein (HABP) and further processed through image analysis to obtain the total intensity of HA in the tissues. (C) The 400 μm cuts *D*_*n*_ were placed inside 2 mL centrifuge tubes for tissue digestion and HA extraction to be quantified using ELISA.

To accurately quantify the HA fluorescence signal, we mitigated the autofluorescence inherent to the tissue samples by acquiring images before (Primary Antibody Image (PAI)) and after (Secondary Antibody Image (SAI)) secondary antibody incubation and washing (Figure 1B). To prevent dehydration during image acquisition, samples were immersed in 1 × PBS. For consistency and reproducibility, fluorescence images were captured using a standardized microscope setup. Mosaic images of the whole sample were taken using a Leica Dmi8 inverted microscope with a 20× magnification dry objective with 0.4 numerical aperture using 100% laser excitation, 200 ms exposure time, and 2 × 2 camera binning. Batch effects and scanner drift were minimized by performing all imaging under fixed conditions. Instrument calibration ensured stable illumination. In addition, intensity normalization was performed to correct for potential shading. After image acquisition, the mosaic images were stitched using a 10% overlap and exported as Tagged Image Files (.tiff) for further processing. To confirm that HABP-b binding was specific to HA, tissues were treated with hyaluronidase (HAase; H1136, Sigma-Aldrich, USA) at pH 7 for 5 h at 37 °C, following the manufacturer’s protocol. Additionally, to further prove its specificity, competitive inhibition of the HABP-b with soluble HA was performed, according to previously published protocols.^20^ Briefly, soluble HA (15MDa, NC1584217, Lifecore Biomedical, USA) at a final concentration of 0.05 mg/mL in the presence of HABP-b (at final concentration of 2.5 μg/mL) for 3 h at RT with gentle shaking before incubation in the tissue sample.

GAG extraction from skin biopsies was performed by digestion of *D*_*n*_ sections (Figure 1C) with papain enzyme following the protocol by van Wijk et al.^21^ with modifications (SI). The GAGs precipitate was resuspended in NaCl 0.9% (w/v) to quantify the HA concentration using an ELISA kit (K-4800, Echelon Biosciences, USA).

Finally, the direct relationship between the total HA of a tissue section to the fluorescence signal intensity (FI) was represented with the following eq (eq 1):

1 $${\left[HA\right]}_{local}=Tk\times \frac{[HA{]}_{total}}{{\left[IF\right]}_{total}}\times {\left[FI\right]}_{local}+\varepsilon$$

where [*FI*]_*total*_ represents the total fluorescence intensity of a *C*_*n*__,*i*_ skin section, [*HA*]_*total*_ is total HA concentration of a *D*_*n*_ skin section (in mg/mL), [*FI*]_*local*_ and [*HA*]_*local*_ are the fluorescence intensity and amount of HA in each pixel of a *C*_*n*__,*i*_ skin section (in arbitrary units (A.U.) and mg/mL, respectively), *Tk* is a thickness normalization term, and ε reflects the residuals between predicted and observed HA values from the calibration curve (SI).

To calculate the total fluorescence intensity ([*FI*]_*total*_), image analysis was carried out with each of the two images obtained for each *C*_*n*__.*m*_ section. The autofluorescence captured after primary antibody incubation (PAI) was subtracted from the image acquired after secondary antibody (SAI) staining. Prior to subtraction, the images were registered using the ImageJ plugin “Landmark-based Correlation” to ensure pixel-wise alignment. The subtraction was then performed using the “Calculator Plus” tool in ImageJ. The resulting image contained only the fluorescence signal attributable to HA. To correct for background signal, the average intensity of nontissue regions (background pixels) was subtracted from the entire image. The total HA-related fluorescence intensity ([*FI*]_*total*_) was then computed by summing all pixel intensities within the segmented tissue area. This value was normalized by the sample volume to yield the FI concentration for the section. The entire workflow is summarized schematically in Figure S1.

For each *C*_*n.m*_ section, the tissue volume was estimated as the product of the segmented area (in pixels) and the section thickness. Otsu thresholding was used to segment the tissue area from the background in the fluorescence images. The resulting volume estimate was used to normalize the total fluorescence intensity, yielding the IF concentration. The final unit for this value is expressed in arbitrary fluorescence units per pixel (AU/px). To generate accurate pixel-wise concentration maps across samples of varying thickness, a normalization factor *Tk* (SI) was applied to account for differences in section thickness. For each *D*_*n*_ section (used for biochemical quantification), volume was calculated assuming the shape of the section is approximately continuous and free of abrupt morphological changes. Thus, the volume (eq 2) was estimated using the average area of the two adjacent thin sections *C*_*n.*__2_ and *C*_*n.*__+1,1_, multiplied by the known thickness (*h*_*D*_*n*__), the thick section (Figure 1A):

$$\mathrm{Volume}\;\left(D_{n}\right)=\frac{\mathrm{Area}\left(C_{n.2}\right)\times \mathrm{Area}\left(C_{n+1.1}\right)}{2}\times h_{D_{n}}$$ 2

By correlating the concentration values obtained from the total fluorescence intensity measured in *C*_*n.m*_ through imaging (FI) and biochemical quantification (via ELISA in *D*_*n*_), a calibration curve was constructed to convert pixel-level fluorescence intensity into absolute HA concentration (Figure 2A). This relationship yielded a robust polynomial regression fit (R^2^ = 0.91), allowing the conversion of fluorescence intensity at each pixel into absolute HA concentration values. Residual analysis demonstrated that the second-order polynomial regression provided a more adequate fit to the data than the linear model (Figure S2). Using this calibration, quantitative spatial maps of HA distribution were generated for tissue sections (Figure 2B). To validate the specificity of the staining, HAase digestion experiments confirmed that the HA signal is selectively lost upon enzymatic degradation of HA (Figure S3A–D). Additionally, sections incubated only with the secondary antibody or with competitive inhibition of the HABP-b with HABP-b confirmed the absence of nonspecific binding to endogenous biotin or other ECM components (Figure S3E,F)

**Figure 2:**
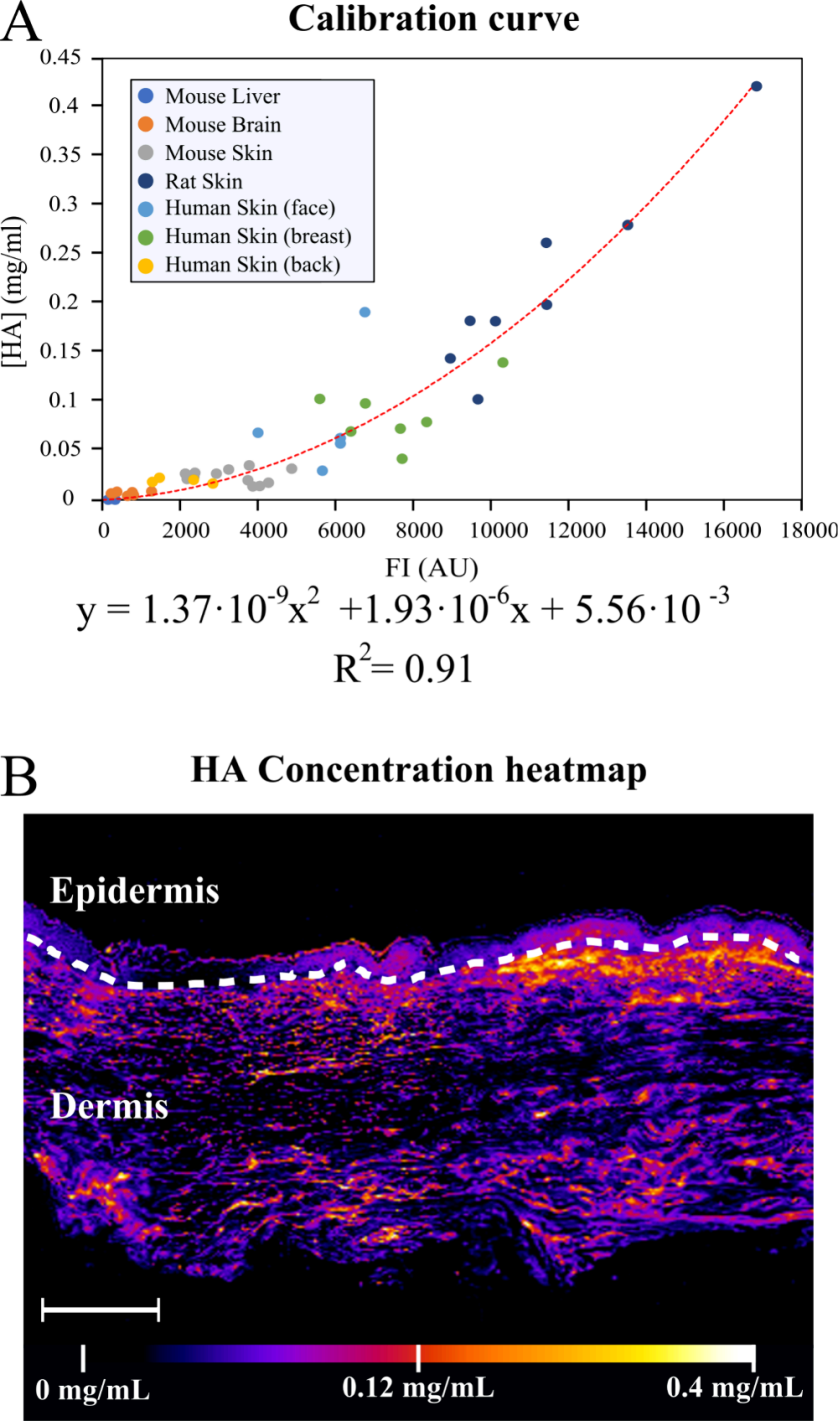
Correlation of the FI signal and HA concentration for the generation of quantitative HA distribution. (A) Polynomial regression for the correlation of HA concentration and fluorescence intensity in each pixel. Each point represents a 400 μm human and rat skin sample. R^2^ = 0.91. (B) Quantitative color map of HA concentration (in mg/mL) of 5 μm human skin samples. Scale bar: 250 μm.

This methodology was applied to assess HA distribution across tissues with known HA presence. In mouse brain sections, HA concentration displayed marked spatial heterogeneity (Figure 3A­(i)). To facilitate analysis, arbitrary radial regions were defined, and pixel-level HA concentrations were represented as density plots (Figure 3B­(i)). These reveal that HA levels were highest near the cortex, HA concentration maps revealed a decreasing gradient from the cortical surface inward (Figure 3C­(i)). This observation is consistent with enrichment of HA in perineuronal nets and other extracellular specializations (9,10,22). Similarly, human (Figure 3A­(ii)) and rat skin (Figure S4) sections show a heterogeneous distribution of HA. Manual segmentation into epidermis, papillary dermis, and reticular dermis reveal a clear separation between the epidermis and deeper layers, with the reticular dermis consistently exhibiting higher HA levels (Figure 3B­(ii)).^13,14^ HA concentration declines gradually with depth (Figure 3C­(ii)). While the relative spatial pattern is consistent, absolute values varied across anatomical sites (Figure S5) and individuals (Figure S6). In contrast, mouse liver sections exhibit a uniform absence of HA (Figure 3A­(iii)), whereas the adjacent gallbladder showed prominent HA accumulation (Figure 3B­(iii), Figure 3C­(iii)).^13^

**Figure 3:**
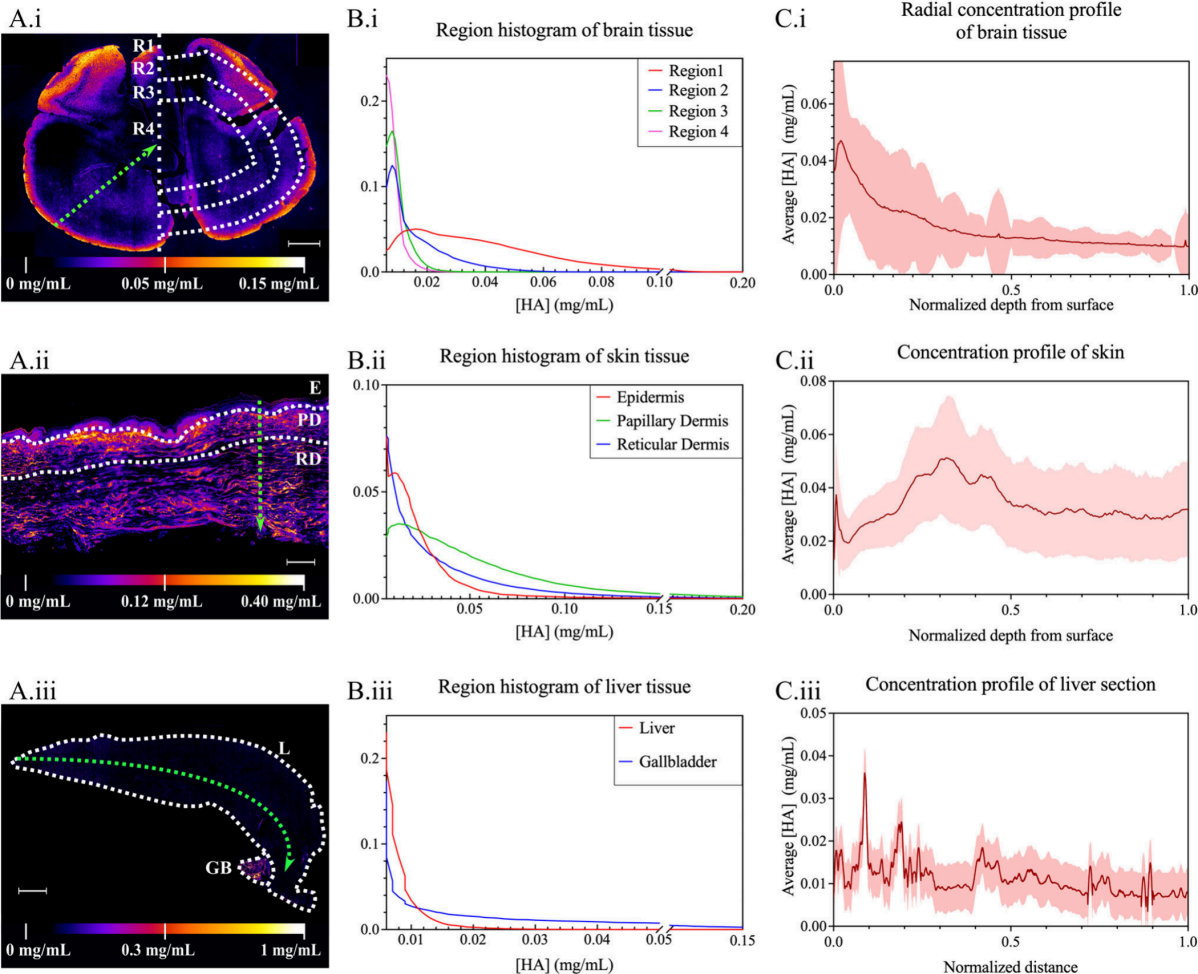
Localization and quantification of HA different tissues. (A) Quantitative color map of HA concentration (in mg/mL) for (i) mouse brain tissue (scale bar; 500 μm), (ii) human skin (scale bar; 250 μm), and (iii) mouse liver and gallbladder (scale bar: 1 mm). (B) Frequency of HA concentration (in mg/mL) for mouse (i) concentric regions of brain tissue, (ii) the epidermis, papillary, and reticular dermis of skin tissue, and (iii) liver tissue and gallbladder. (C) Concentration profile of HA concentration (in mg/mL) in (i) radial distribution in brain tissue, (ii) linear distribution in the epidermis, papillary and reticular dermis of skin tissue and (iii) linear distribution in the liver tissue. Green arrows show the direction of the represented concentration profile. Notation: R1 (Region 1); R2 (Region 2); R3 (Region 3); R4 (Region 4); E (epidermis); PD (papillary dermis); RD (reticular dermis); L (liver) and GB (gallbladder).

Finally, to assess reproducibility, both intraslide and interslide variability were quantified. Technical replicates (duplicate sections from the same block) consistently showed intraslide coefficients of variation (CVs) of approximately 25%, while interslide biological replicates (different individuals per group) yielded CVs in the range of 44.00 ± 17.20% (95% CI) Table S1. Intraclass correlation coefficients (ICCs) further confirmed good reproducibility (ICC = 0.904 ± 0.041% (95% CI)) within samples Table S2.

By enabling absolute quantification, this approach permits direct comparison of HA concentrations across tissues, individuals, and experimental conditions. This capability extends beyond qualitative or relative analyses, facilitating reproducible benchmarking of HA abundance in both physiological and pathological contexts. In skin, HA exhibits a differential distribution aligned with well-defined anatomical structures. The higher HA levels observed in the reticular dermis compared to the epidermis are consistent with the established distribution of HA within the extracellular matrix.^14,17^ In contrast, the organization of brain regions is considerably more complex.^22^ In this study, brain data are presented using a simplified spatial representation to validate the methodology. Nonetheless, this approach can be applied in future investigations specifically designed to examine the biological relevance of HA distribution across distinct brain areas. Such studies, incorporating anatomically guided segmentation and targeted experimental designs, will enable a more detailed assessment of region-specific HA organization and its functional implications. Regarding the distribution and organization of HA in mouse liver sections, the sharp contrast in HA concentration between liver and gallbladder regions is noteworthy. The biological relevance of this difference likely relates to the viscoelastic and hydrating properties of HA, which support mechanical resilience, lubrication, and volume regulation. Such characteristics are consistent with the gallbladder’s physiological role as a distensible reservoir for bile storage and release.^23^ The ability to quantify these tissue-specific patterns highlights the potential of absolute HA mapping to connect extracellular matrix composition with organ function.

The findings from this study provide a framework for determining absolute HA concentrations at defined spatial locations within diverse tissue types. This capability is critical for several applications: (i) the development of physiologically relevant in vitro models that better mimic native tissue microenvironments; (ii) a more precise understanding of the role of HA in disease progression, including its associations with cancer prognosis and treatment outcomes; and (iii) the potential use of HA distribution as a complementary diagnostic or prognostic biomarker in various pathologies. Notably, the method has been optimized for HA, a particularly challenging molecule to quantify due to its nonimmunogenic and nonproteinaceous nature, as it is a polysaccharide rather than a protein and lacks stable epitopes for conventional immunochemical detection. In addition, HA is highly dynamic and susceptible to endogenous enzymatic and oxidative degradation,^24–26^ a factor especially relevant in our study since the analyzed tissues were obtained from deceased donors. Precise post-mortem intervals and fixation times were not available for human donor tissues, and therefore, some degree of HA degradation prior to fixation cannot be excluded. This factor is especially relevant given the short half-life of HA in skin and other tissues.^27–29^ Nevertheless, because the calibration curve is derived from paired sections of the same tissue block, any degradation occurring prior to fixation is internally accounted for, ensuring robust relative quantification and reliable spatial mapping. Furthermore, several methodological limitations should be acknowledged. First, our workflow was optimized for paraffin-embedded tissues, which ensures broad applicability in pathology and provides superior structural preservation compared with frozen sections.^30^ While HABP-based staining is also compatible with frozen samples, systematic benchmarking of the calibration workflow in that context remains to be performed. In this regard, fixation parameters such as temperature, blocking conditions, and incubation times must be carefully controlled to prevent HA degradation,^19^ and complete paraffin removal is essential to ensure optimal accessibility for HABP binding.^31,32^ No antigen-retrieval steps are required, as HA is a polysaccharide rather than a protein.^33^ Second, HABP affinity may vary with HA chain length or chemical modifications,^34,35^ potentially affecting staining intensity. Third, the present method provides absolute concentration maps but does not capture HA molecular weight distribution, which is critical for bioactivity.^36,37^ Future refinement will involve validating frozen tissue compatibility, cross-validating HABP binding across HA size variants, and integrating molecular weight–sensitive analytical assays. While this study focused on healthy tissues to establish and validate the workflow, applying the method to pathological contexts in future works—such as tumor microenvironments or fibrotic tissues—will help reveal biologically relevant differences in HA distribution. Combining molecular weight–sensitive techniques with analyses of annotated human and animal cohorts will allow systematic assessment of how donor factors and ECM remodeling (e.g., fibrosis or edema) influence HA spatial organization and quantitative mapping.^38,39^

To place these findings in context, our results should be interpreted in light of previous efforts to visualize HA in tissues, many of which relied on histochemical, biochemical, or semiquantitative fluorescence methods.^13–15,22^ While these approaches provided valuable qualitative insights, they were limited by relative measurements, restricted reproducibility, and a lack of pixel-level resolution. By integrating HABP-based fluorescence imaging with ELISA calibration from paired sections, the methodology presented here offers an accessible workflow for absolute HA quantification in standard paraffin-embedded samples. This advance enables reproducible comparisons of HA concentrations across tissues, individuals, and experimental conditions.

Recent developments in extracellular matrix (ECM) imaging and quantification—such as second harmonic generation (SHG) microscopy for collagen and MS imaging for proteoglycans—have substantially advanced our understanding of ECM organization and dynamics.^40–43^ However, these techniques fail to provide both absolute quantitative and highly spatially resolved information across a broad range of ECM components, particularly glycosaminoglycans such as HA, whose nonprotein, highly hydrated nature makes them difficult to detect and quantify with label-free or MS-based modalities. Despite inherent challenges in HA detection, our HABP-based workflow enables absolute, spatially resolved quantification of HA, yielding robust quantitative data that can be readily integrated with measurements of other ECM components, including collagen I, fibronectin, or elastin. Importantly, the methodology relies only on standard laboratory equipment—such as a fluorescence microscope—and does not require specialized instrumentation, making the workflow broadly accessible and scalable for histological studies. Overall, this pipeline provides a practical and reproducible tool for in situ spatial quantification of ECM components in paraffin-embedded tissues, extending the ECM imaging repertoire and supporting comprehensive analyses of tissue organization, remodeling, and function.

## Supplementary Material



## Notes

Experimental details for tissue processing, fluorescence staining, tissue digestion and calibration of fluorescence intensity to hyaluronic acid concentration, and data reliability (PDF)

## Notes

**Ethics Statement**: For this study, human skin biopsies of 2–3 cm^2^ were obtained from deceased organs of three different donors after informed written consent following Spanish laws in organ and tissue donation from Banco de Sangre del Centro Comunitario de Transfusión del Principado de Asturias (CCST), Asturias, Spain. On the other hand, rat (5-month-old, WISTAR) and mouse (5-month-old, C57BL/6) brain, skin and liver biopsies were collected from discarded animals at Universidad Carlos III de Madrid and Centro de Investigaciones Energéticas, Medioambientales y Tecnológicas (CIEMAT). Three independent animals—2 males and 1 female—were analyzed for each tissue sample. The study was conducted in accordance with the Declaration of Helsinki and approved by the Institutional Ethics Committee of Universidad Carlos III de Madrid (Protocol code: CEI_24_05_MELANOMA_CHIP; Date of approval: 10 April 2024).
