# Supplementary material for: Absolute Quantification and Spatial Mapping of Hyaluronic Acid in Histological Tissue Sections
Source: ACS Meas Sci Au. 2025 Nov 11;6(1):21–7. doi: 10.1021/acsmeasuresciau.5c00138 (PMC12921588; doi:10.1021/acsmeasuresciau.5c00138)
Supplement: Supplementary file 1 [file tg5c00138_si_001.pdf]

Supporting Information

Absolute Quantification and Spatial Mapping of Hyaluronic Acid in Histological Tissue Sections

Cristina Quílez[1,2,‡], Jorge González-Rico[3,‡], María Luisa López-Donaire[3], Nuria Gago-López[4], Arrate Muñoz-Barrutia[5,6,*], Diego Velasco[1,2,*]

1: Bioengineering Department, Universidad Carlos III de Madrid, 28911, Leganés, Spain; 2: Fundación Instituto de Investigación Sanitaria de la Fundación Jiménez Díaz, 28040, Madrid, Spain; 3: Continuum Mechanics and Structural Analysis Department, Universidad Carlos III de Madrid, 28911, Leganés, Spain;4: Melanoma group. Molecular Oncology Program. Spanish National Cancer Research Center (CNIO), 28029, Madrid, Spain; 5: Neuroscience and Biomedical Sciences Department, Universidad Carlos III de Madrid, 28903, Getafe, Spain; 6: Instituto de Investigación Sanitaria Gregorio Marañón, 28009, Madrid, Spain.

Experimental Details

Tissue processing

Tissue samples were fixed for 24 hours using formalin-free tissue fixative (A5472, Sigma Aldrich, USA) to prevent HA loss. Then, samples were washed for 15 minutes in running tap water and maintained for an extra 24 hours in 1×PBS at 4ºC. For paraffin embedding, tissues were processed in the Tissue Processor (ASP 300S, Leica Biosystems, USA). Briefly, samples were incubated overnight in ethanol 70% at room temperature (RT), followed by two consecutive 15 minutes incubation at 37ºC in ethanol 70%, two consecutive 45 minutes incubation at 37ºC in ethanol 90%, three consecutive 45 minutes incubation at 37ºC in ethanol 100%, three consecutive 30 minutes incubation at 37ºC in 100% X-Free solvent (06-1305Q, Bio-Optica, Italy), two consecutive 45 minutes incubation in paraffin (CA-09-5658, Histo-Comp, Casa Álvarez, Spain) at 65ºC and an overnight incubation in paraffin at 65ºC. Finally, tissue sections were embedded in paraffin to obtain the tissue blocks.

Fluorescence Staining

The fluorescence staining of HA within tissue sections started with the deparaffinization of the $C_{n,m}$ samples (**Figure 1b**). Namely, the samples were fixed to the slides by incubating at 65ºC for 15 minutes. After which, they were deparaffined following the subsequent immersions in: two consecutive 100% X-Free solvent (06-1305Q, Bio-Optica, Italy) for 10 minutes, 37ºC with gentle shaking, 50% X-Free solvent/100% ethanol, two consecutive 100% ethanol, two consecutive ethanol 95%, ethanol 70%, ethanol 50%, distilled H_2O (v/v) and three consecutive 1×PBS, all for 5 minutes at RT.

Next, the samples were incubated in 3% (w/v) PBS-Bovine Serum Albumin (BSA) for 45 minutes at RT. For HA fluorescence staining, the samples were incubated with Biotinylated Hyaluronic Acid Binding Protein (HABP-b) (AMSBio, USA; 1:100 dilution in 1× PBS-BSA 3%) overnight at 4ºC and washed three times with 1×PBS. Then, for secondary antibody staining, samples were incubated with Alexa Fluor 488-Streptavidin conjugate (S11223, ThermoFisher, USA; diluted 1:1000 in 1×PBS) for 1 hour at RT and again washed three times in 1×PBS.

Tissue digestion for HA quantification and ELISA assay

Glycosaminoglycans (GAGs) extraction from skin biopsies was performed by digestion of D_n sections (**Figure 1a**) with papain enzyme following the protocol by van Wijk et al. (*19*) with modifications. First, for sample deparaffination, D_n tissue sections were introduced in consecutive 100% X-Free solvent (06-1305Q, Bio-Optica, Italy) baths for 15 minutes at 4ºC and then centrifuged for 5 minutes at 14,000×g. Tissue sections were consecutively washed in 100%-, 96%-, 70%-, 50%- and 30% ethanol (v/v) in 1×PBS for 3 minutes at RT. Then, samples were rinsed in tap water and rehydrated in 1×PBS for 30 minutes at RT. Then, for tissue digestion, 20U of papain enzyme (P3125, Sigma-Aldrich, USA) diluted in digestion buffer pH 6.5 (50mM Na_3PO_4 (939056, Sigma-Aldrich, USA), 2mM cysteine (30095, Sigma-Aldrich, USA) , and 2mM ethylenediaminetetracetic acid (ED2SS, Sigma-Aldrich, USA)) were added to each previously deparaffinized D_n section and incubated at 65ºC for 16 hours after which it is inactivated for 1 hour at 95ºC. Finally, GAGs were precipitated with 3% (w/v) NaCl and two volumes of acetone/methanol (70:30) on ice with gentle shaking. Samples were centrifuged at 1600*g* for 15 minutes and dried at RT for complete water evaporation and stored at -20ºC. The GAGs precipitate was resuspended in NaCl 0.9% (w/v) and analyzed using an ELISA kit (K-4800, Echelon Biosciences, USA) for HA quantification following manufacturer's protocol.

Calibration of Fluorescence Intensity to Hyaluronic Acid Concentration

The direct relationship between the total HA of a skin section and the fluorescence signal intensity (FI) can be described as follows:

$$FI_{total} \rightarrow HA_{total}$$

$$FI_{local} \rightarrow HA_{local},$$

Here, FI_{total} is the total fluorescence intensity measured in a section a $C_{n.i}$, HA_{total} is the total HA content in the paired section D_n processed for ELISA quantification. FI_{local}

and HA_{local} refer to the fluorescence intensity and HA concentration at each pixel within the $C_{n.i}$ section, respectively. Assuming a linear relationship and spatial homogeneity of signal conversion, HA_{local} can be approximated by normalizing the pixel intensity to the total signal:

$$HA_{local} = \frac{FI_{local} \times HA_{total}}{FI_{total}}$$

This equation enables pixel-wise mapping of HA concentration based on the global biochemical quantification from the ELISA assay. To express HA values as concentrations, we divide the total volume of the respective sections: D_n for global (ELISA-based) measurements and $C_{n.i}$ for local (imaging-based) measurements. The local HA concentration $[HA]_{local}$ can then be estimated as:

$$[HA]_{local} = Tk \times \frac{[HA]_{\ total}}{[IF]_{total}} \times [FI]_{local} + \varepsilon,$$

where $[HA]_{\ total}$ is the total HA concentration obtained by ELISA in section D_n, $[FI]_{total}$ is the total fluorescence intensity in the corresponding imaging section $C_{n.i}$, $[FI]_{local}$ is the fluorescence intensity at each pixel, and ε, represents the random error. This relationship forms the basis of the calibration curve linking fluorescence signal to HA concentration using skin sections of 5 µm ($C_{n.i}$). When applying this calibration to tissue samples with different thicknesses, a thickness normalization factor Tk is introduced to account for such variability:

$$Tk = \frac{h_{\ C_{n.i}}}{h_{\ i}}$$

where $h_{\ C_{n.i}}$ is the thickness of the reference section used for calibration, and $h_{\ i}$ is the thickness of the sample under analysis. This correction ensures accurate quantification across samples of varying thickness.

Data reliability

All imaging was performed under fixed and standardized microscope conditions (identical laser power, exposure time, and camera binning across all samples) to minimize batch effects and scanner drift. Daily instrument calibration ensured stable illumination, and mosaic acquisition settings were kept constant. In addition, intensity normalization was performed across mosaics to correct for potential shading. No color drift was observed, as only a single fluorescence channel was used.

Several technical and biological replicates were analyzed to ensure reliability:

- **Technical replicates**: Duplicate 5 µm sections were cut from the same tissue block and processed independently (staining, imaging, and quantification) per slide. Multiple slides (2x cuts) were obtained per sample, and multiple samples were considered per tissue and species.

- **Biological replicates**: Skin, brain, and liver/gallbladder samples from n = 3 human donors (2 males and 1 female) and n = 3 animals per rat (5-month-old, WISTAR) and mouse (5-month-old, C57BL/6) (2 males and 1 female). For skin tissue, different body locations were analyzed: 1) back skin in rats and mice, and 2) breast, face, and back skin in humans.

Across all tissue types, intra-slide CVs were around the value 25.85±6.65 (95% CI) while inter-slide CVs within the same sample were in the range of 22.416±7.64 (95% CI), reflecting the robustness of the capability of the method to locate HA. Regarding the inter-sample CV, the variation within the same tissue but different samples was 44.00±17.20(95% CI), reflecting expected biological variability. In addition, we computed intraclass correlation coefficients (ICC), which indicated good reproducibility (ICC values for FI measurements = 0.904±0.041 (95% CI); ICC values for [HA] measurements = 0.845±0.096 (95% CI)) within samples. The ICC values were greatly reduced within tissue and within species measurements, again reflecting the expected biological variability.

Together, these analyses confirm that the workflow is robust and reproducible across both technical and biological replicates.

Residual analysis of regression curves is represented in figure S2. Root Mean Squared Error (RMSE) values were computed for both linear and second order polynomial regressions.

$$RMSE_{lineal} = 0.0414$$

$$RMSE_{cuadratic} = 0.0266.$$

Table S1: Intra-slide, inter-slide and inter-sample Coefficients of Variation (CVs)

	[FI] from imaging
Intra-slide	25.85±6.65 (95% CI)
Inter-slide (within sample)	22.416±7.64 (95% CI)
Inter-sample (within tissue)	44.00±17.20 (95% CI)

Table S2: Interclass Correlation Coefficients (ICC) for FI and HA concentration measurements within samples, tissues and species.

	[FI] from imaging	[HA] from ELISA
Within sample	0.904±0.041 (95% CI)	0.845±0.096 (95% CI)
Within tissue	0.611±0.117 (95% CI)	0.802±0.082 (95% CI)
Within species	0.418±0.115 (95% CI)	0.786±0.036 (95% CI)

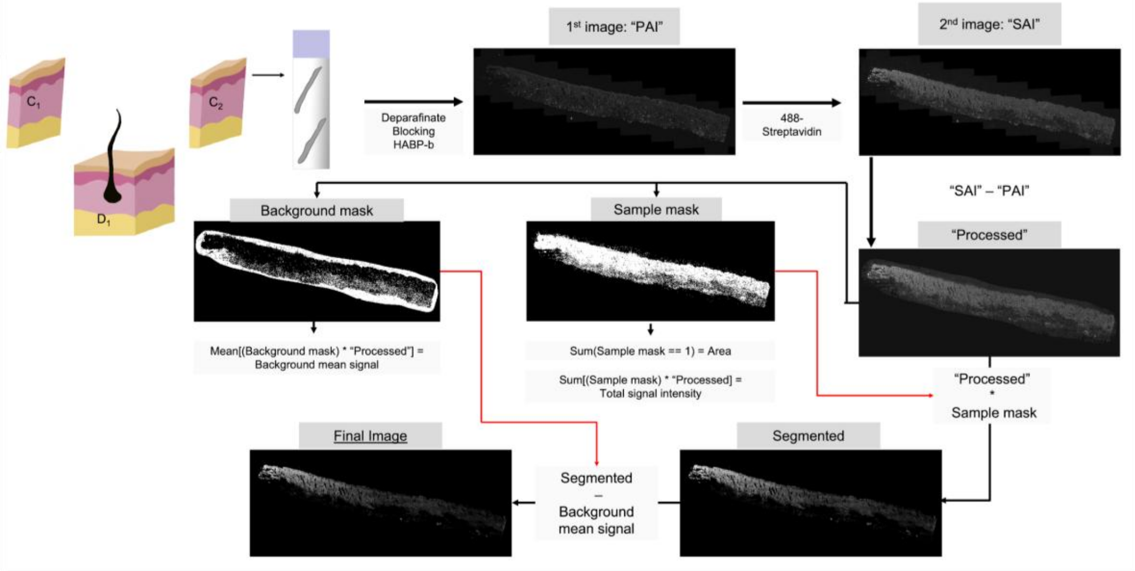

Figure S1: Image processing workflow for quantification of HA-specific fluorescence signal. Schematic representation of the pipeline used to obtain background-corrected HA fluorescence signal from paraffin-embedded tissue sections. Adjacent thin sections C_1 and C_2 are processed for imaging, while the thick section D_1 is used for ELISA quantification. After deparaffinization and blocking, the first fluorescence image ("PAI") is acquired after incubation with HABP-b. The second image ("SAI") is acquired after Alexa Fluor 488-Streptavidin staining. The HA-specific signal is obtained by subtracting "PAI" from "SAI" to generate the "Processed" image. Sample and background masks are applied to extract total and background signals. The final image is obtained by subtracting the mean background signal from the segmented processed image. Area and intensity values are then used to calculate total signal and concentration.

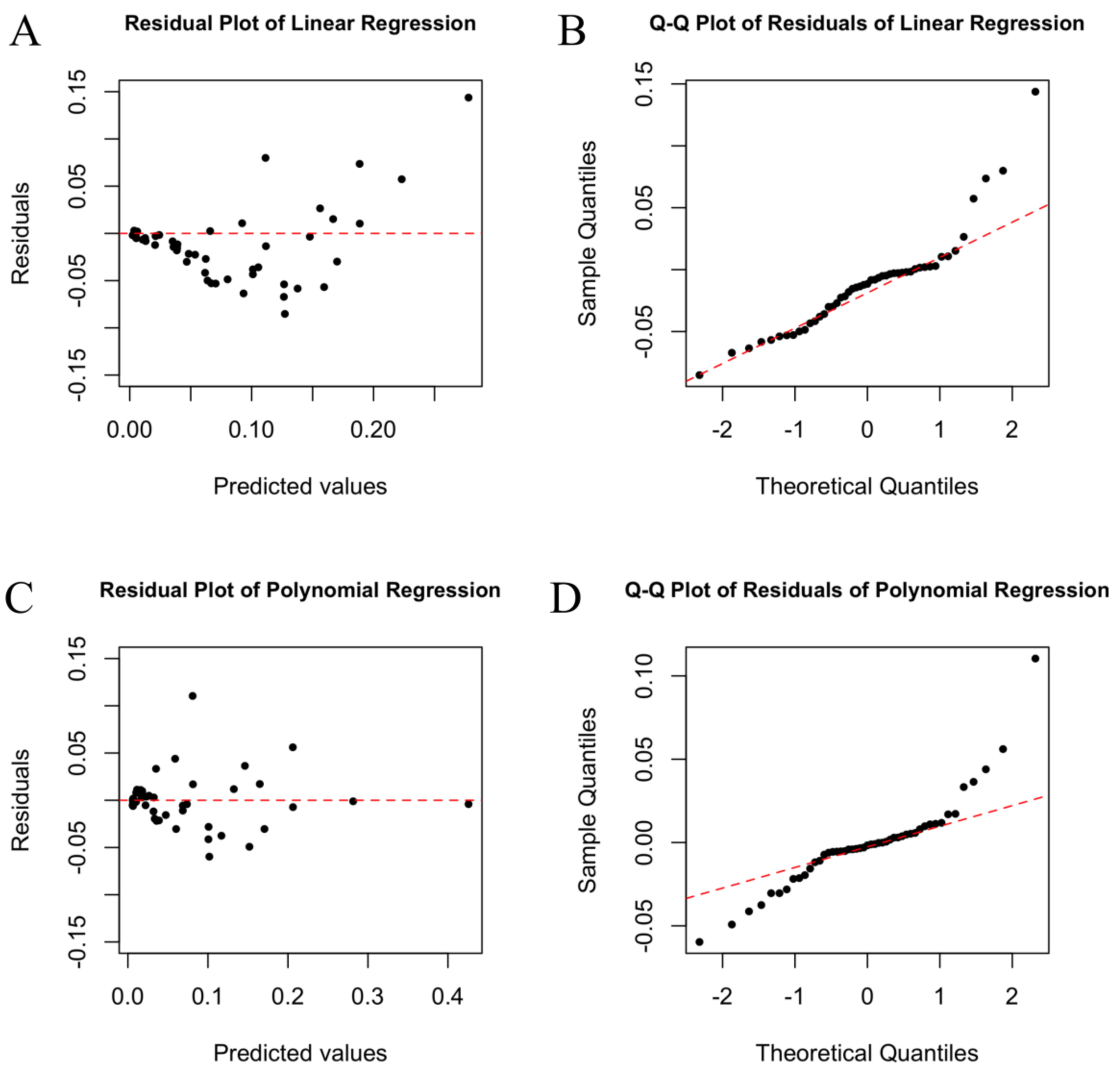

Figure S2: Residual diagnostics for linear and second-order polynomial regression models. (A) Residuals versus fitted values for the linear regression. (B) Q–Q plot of residuals for the linear regression. (C) Residuals versus fitted values for the second-order polynomial regression. (D) Q–Q plot of residuals for the polynomial regression.

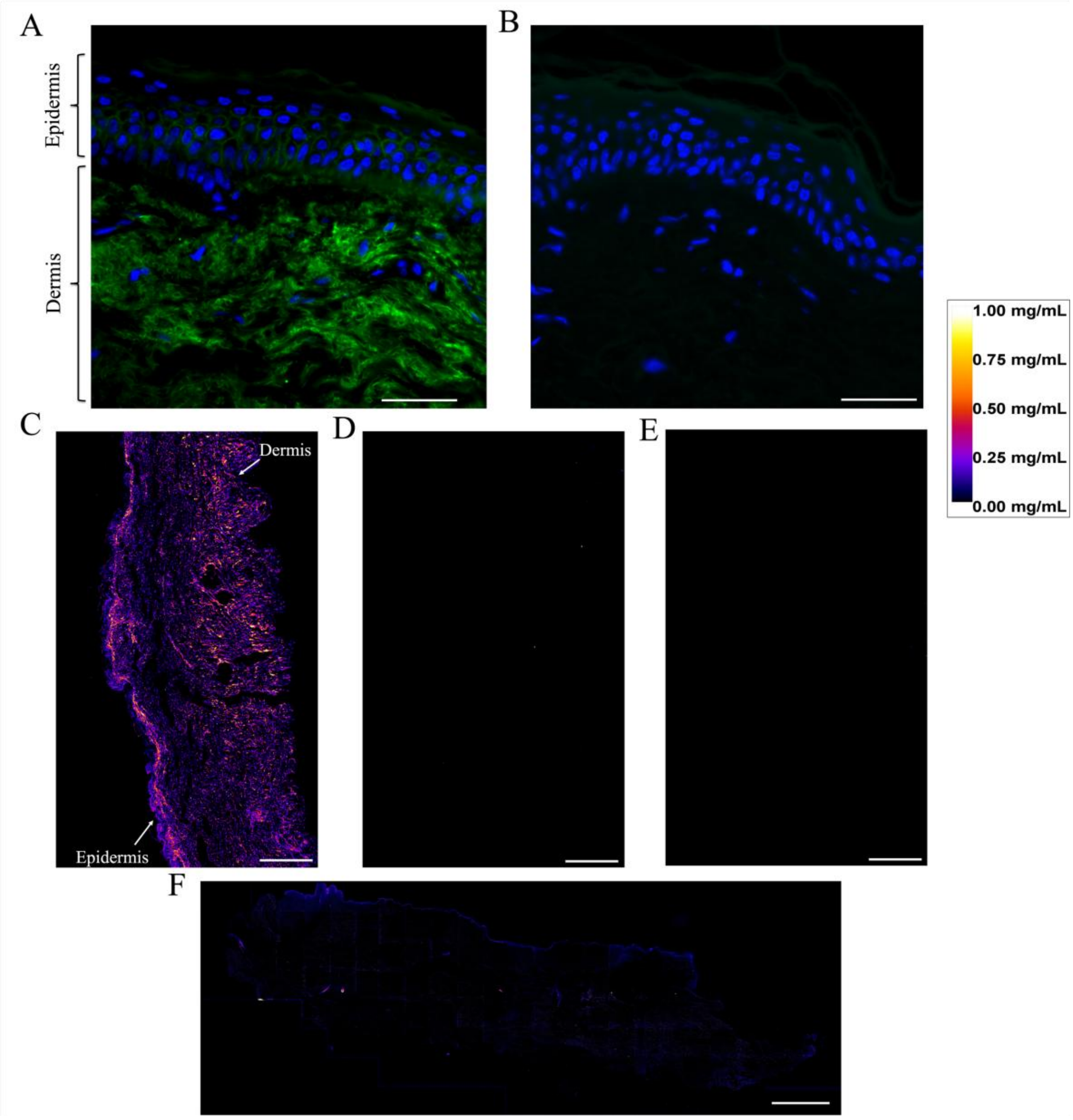

Figure S3: BP specificity test. (**A**) Immunofluorescence image showing HA localization (green) in human skin sections in control conditions and (**B**) after Hyaluronidase enzyme (HAase) digestion. Cell nuclei are stained in blue DAPI. Scale bar 50 µm. (**C**) Color-map of HA concentration (in mg/mL) for human skin samples under control conditions. (**D**) Color-map of [HA] after HAase digestion. (**E**) Color-map of [HA] localized with only secondary antibody incubation. (**F**) Color-map of [HA] after competitive inhibition of the HA-HABPb. Scale bar: 200 µm.

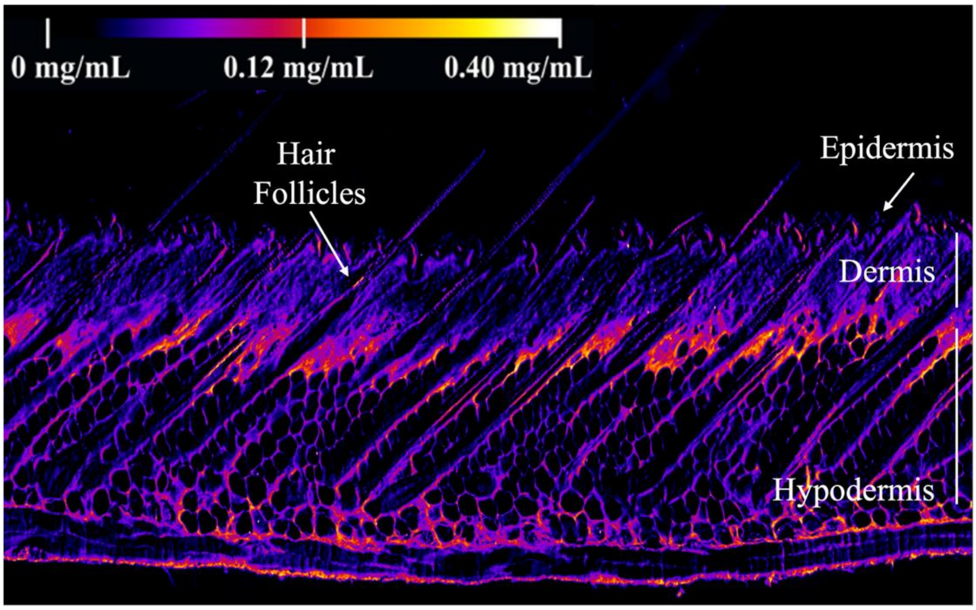

Figure S4: Quantitative mapping of HA concentration in rat skin. Heatmap of hyaluronic acid (HA) concentration (mg/mL) in a representative skin section, showing higher HA levels in the hypodermis and near the hair follicles.

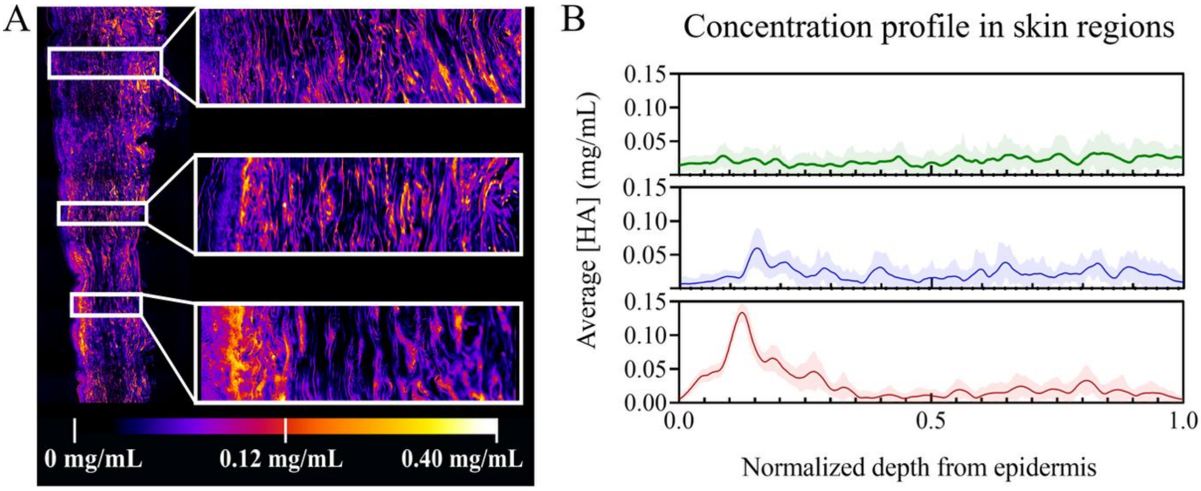

Figure S5: Quantitative mapping and depth profile of HA concentration in human skin. (A) Heatmap of hyaluronic acid (HA) concentration (mg/mL) in different areas of the same tissue (white squares) showing differential HA levels in the reticular and papillary dermis compared to the epidermis depending on the region. (**B**) Average HA concentration profile as a function of normalized depth from the skin surface corresponding to different areas of the same tissue (white squares).

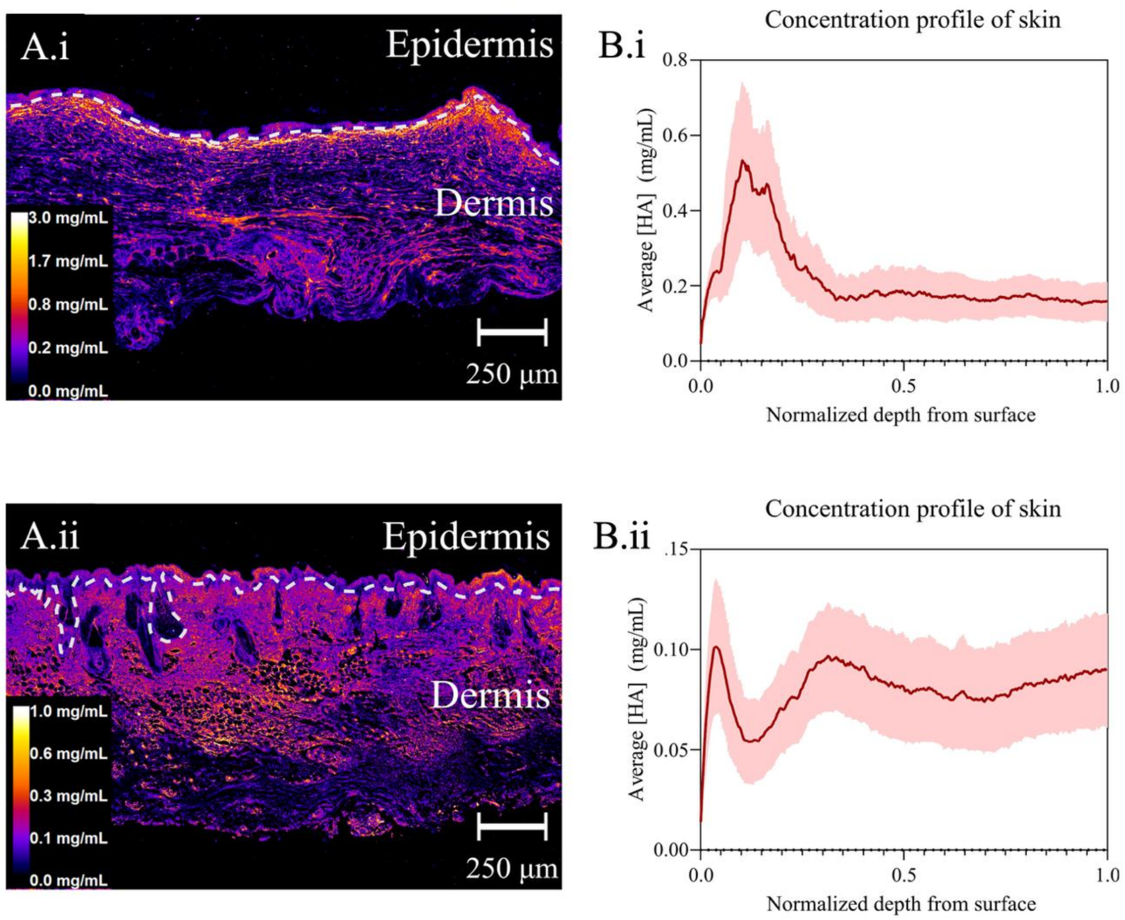

Figure S6: Quantitative mapping and depth profile of HA concentration in human skin. (**A)** Heatmap of hyaluronic acid (HA) concentration (mg/mL) in (**i**) a representative skin section, showing higher HA levels in the reticular dermis compared to the epidermis and (**ii**) a second representative skin section with lower overall HA content. (**B**) Average HA concentration profile as a function of normalized depth from the skin surface in a (**i**) first representative skin section and (**ii**) second representative skin section; shaded area indicates standard deviation across pixels at each depth. Scale bars: 250 μm.
